# Revolutionizing nephrocalcinosis treatment: IL‐10 engineered macrophages as a novel therapeutic approach

**DOI:** 10.1002/btm2.70047

**Published:** 2025-07-10

**Authors:** Wenlei Zhao, Kailong Wang, Hairui Chen, Junnan Xu, Lequan Wen, Yan Wu, Weihao Chen, Haitao Liu, Yanzhong Liu, Jiqing Zhang, John C. Lieske, Luo Zhang, Xu Zhang, Haixing Mai

**Affiliations:** ^1^ Department of General Surgery The First Medical Center, Chinese PLA General Hospital Beijing China; ^2^ Department of Urology The Third Medical Center, Chinese PLA General Hospital Beijing China; ^3^ The Fifth Clinical School of Medicine Anhui Medical University Hefei China; ^4^ Medical School of Chinese PLA Beijing China; ^5^ School of Medicine Nankai University Tianjin China; ^6^ Department of Urology Beijing Anzhen Hospital, Capital Medical University Beijing China; ^7^ Department of Internal Medicine, Division of Nephrology and Hypertension Mayo Clinic Rochester Minnesota USA; ^8^ Department of Laboratory Medicine and Pathology Mayo Clinic Rochester Minnesota USA; ^9^ Research Center of Bioengineering, the Medical Innovation Research Division Chinese PLA General Hospital Beijing China

**Keywords:** calcium oxalate, interleukin‐10, macrophage transplantation, nephrocalcinosis

## Abstract

Nephrocalcinosis provides a nidus for stone formation and is strongly linked to renal injury and chronic kidney disease. Although the crucial role of macrophages in the formation and progression of calcium oxalate (CaOx) crystals has long been widely recognized, finding effective immunotherapies for nephrocalcinosis remains a challenge. In this study, we described an innovative macrophage‐based method that delivers interleukin‐10 (IL‐10) into the kidney, reduces the deposition of CaOx crystals, and alleviates renal injury in a mouse model of glyoxylate‐induced CaOx. Compared with recombinant IL‐10 direct injection, the macrophage‐based method has the advantages of biocompatibility and sustaining action. We found that the transplantation of engineered macrophages via the tail vein significantly reduced the volume of crystals in the kidney, thereby alleviating the kidney injury caused by crystal. In mechanistic studies, IL‐10‐secreting macrophages inhibited crystal formation and promoted crystal clearance by promoting macrophage M2 polarization, exerting a protective effect on renal tissue. Our data suggest that macrophage‐based delivery of IL‐10 to the kidney can be a potential treatment method for nephrocalcinosis.

**Translational Impact Statement**

In our current study, we overexpressed anti‐inflammatory IL‐10 in macrophages and introduced them into a glyoxylate‐induced CaOx mouse model. Using macrophages' homing ability, the Mφ‐IL10 were selectively directed toward the CaOx crystals. By modulating the local inflammatory microenvironment, Mφ‐IL10 reduced the burden of crystals and mitigated associated renal injury. These findings support Mφ‐IL10 as an immunomodulatory approach for promoting crystal phagocytosis and attenuating renal damage, offering prospects for nephrocalcinosis treatment.

## 1 INTRODUCTION

Nephrocalcinosis is closely associated with kidney stones, often coexisting and sharing pathogenic mechanisms driven by urinary supersaturation of calcium and other ions.^1^, ^2^ It provides a nidus for stone formation and is strongly linked to renal injury and chronic kidney disease (CKD).^3^ Population studies show individuals with nephrocalcinosis have a significantly increased risk of CKD progression and reduced glomerular filtration rate.^4^ The calcium deposits directly damage renal tubules and interstitium, triggering inflammation and accelerating fibrosis.^5^, ^6^ The activation of the NLRP3 inflammasome pathway by CaOx crystals indicates that inflammatory factors play a role in the process of kidney injury related to nephrocalcinosis.^7^ When CaOx crystals deposit in the kidney, they can trigger the NLRP3 inflammasome, which in turn activates a series of inflammatory responses. In summary, nephrocalcinosis contributes to both kidney stone formation and renal damage, highlighting the importance of early intervention to prevent CKD progression.

Macrophage involvement in calcium‐containing crystals in the kidney was first reported by de Water et al. in 1999,^8^ who observed that macrophages migrate to crystal deposition sites and engulf the crystals. Subsequent research has well established the role of macrophages in balancing CaOx crystals formation and clearance, as well as in modulating inflammatory responses.^9^ Macrophage accumulation and migration in the kidney are strongly correlated with structural damage and kidney dysfunction.^10^ Given the M1 pro‐inflammatory and M2 anti‐inflammatory polarization, this fine‐tuning effect is predictable. Generally, M2 macrophages can engulf crystals more efficiently and inhibit kidney injury caused by excessive inflammation compared to M1 macrophages, a characteristic that makes them promising for therapeutic intervention.^11^ Meanwhile, other certain inflammatory factors, such as NLRP3 and SIRT3, have been shown to prevent calcium deposition and effectively remove crystals in animal models.^7^, ^12^ However, there is still no viable immunotherapy based on macrophages for nephrocalcinosis treatment.

Interleukin‐10 (IL‐10) is a well‐known anti‐inflammatory cytokine that promotes M2 macrophage polarization and is produced by nearly all subsets of leukocytes.^13^, ^14^ CaOx, rather than potassium or ZnOx, induces M1 differentiation in human monocytes and primary human mononuclear cells, secreting cytokines and chemokines.^15^ However, ongoing polarization processes may damage normal tissues and organs.^16^ In contrast, high levels of IL‐10 induce macrophage M2 polarization, which is involved in immune regulation, inflammation inhibition, and the stimulation of new extracellular matrix (ECM) formation through angiogenesis and tissue remodeling.^17^

The short in vivo half‐life (1–2 min) of IL‐10 is a major obstacle to its application.^18^ To enhance IL‐10 enrichment in the kidney, several novel drug delivery systems have been reported. Soranno et al. used self‐assembled, injectable Dock and Lock hydrogels to deliver IL‐10 to the kidneys of mice with unilateral ureteral obstruction (UUO), which remained in the mice for 30 days and significantly reduced inflammatory cell infiltration and tissue fibrosis.^19^ In this study, we constructed a macrophage model for continuous IL‐10 secretion and found that macrophage transplantation had a significant therapeutic effect on glyoxylic acid‐induced nephrocalcinosis in mice. Mice transplanted with IL‐10‐secreting macrophages showed a substantial reduction in crystal volume and tubular damage. This study offers a practical strategy for the application of IL‐10 and macrophage transplantation in the treatment of nephrocalcinosis.

## 2 MATERIALS AND METHODS

### 2.1 CaOx mice model

All animal experiments were conducted in strict compliance with the guidelines and protocols approved by the Animal Ethics Committee of the PLA General Hospital. Healthy male C57BL/6 mice, aged 6–8 weeks, were procured from Beijing Sinogene Biotechnology Co., Ltd. (Beijing, China). The mice were housed up to three per cage at a temperature of 25 ± 1°C with a 12‐h light/dark cycle and provided with ad libitum access to food and water. Following a one‐week acclimation period, the mice were randomly assigned to experimental groups (*n* = 5). The calcium oxalate (CaOx) mouse model was induced using the standard glyoxylate method. Specifically, the CaOx mouse models were induced by intraperitoneal injection of 75 mg·kg^−1^·day^−1^ glyoxylate (dissolved in phosphate‐buffered saline (PBS; pH 7.4)) for 6 consecutive days, while control group mice received an equivalent volume of PBS. Engineered macrophages (2.5 × 10^5^ cells/mouse), recombinant IL‐10 (rIL‐10, 50 μg/kg) or an equivalent volume of PBS were administered to the mice via the tail vein 10 h after the initial glyoxylate injection on day 1. On day 7, the mice were euthanized under controlled isoflurane anesthesia and subsequently underwent cardiac perfusion.

### 2.2 Cell culture

Mouse monocyte–macrophage cell line was purchased from Pricella (Wuhan, China), and mouse renal tubular epithelial cell line TCMK‐1 was purchased from QuiCell (Shanghai, China). RAW264.7 and TCMK‐1 cells were cultured and maintained in DMEM and MEM (Gibco, USA), respectively, supplemented with 10% fetal bovine serum (Evergreen, China) and 1% penicillin–streptomycin (Gibco, USA) at 37°C/5% CO2.

### 2.3 Establishing engineered macrophages

The lentiviral constructs expressing murine IL‐10 were cloned into the pCDH vector (System Biosciences, SBI). These lentiviral vectors were transfected along with packaging plasmids into 293 T cells, and the resultant viral particles were harvested and utilized to infect RAW264.7 cells. Subsequent selection of successfully infected cells was performed by culturing in a medium supplemented with 5 μg/mL puromycin for a period of 2 days.

### 2.4 Preparation of calcium oxalate monohydrate (COM)

COM crystals were prepared following a previously described protocol. In summary, 10 mM CaCl_2_ was mixed with 10 mM Na_2_C_2_O_4_ in a 10 mM Tris–HCl buffer solution (pH 7.4) containing 90 mM NaCl to achieve final concentrations of 5 mM CaCl_2_ and 0.5 mM Na_2_C_2_O_4_. The mixture was then incubated at room temperature overnight. The resulting COM crystals were harvested through filtration, sterilized by autoclaving, and subsequently utilized in the following experiments.

### 2.5 Cell adhesion assay

Engineered macrophages were cultured in a 6 cm dish to 80% confluence, after which the medium was replaced, and the cells were further cultured for 48 h. The supernatant was collected for inducing RAW264.7 cell polarization. To assess the adhesive capacity of induced RAW264.7 cells, they were cultured in DMEM containing 100 μg/mL calcium oxalate monohydrate (COM) for 24 h. For TCMK‐1 cells, TCMK‐1 cells were seeded in a 24‐well plate, and RAW264.7 cells were placed in the upper chamber for co‐culturing. After 24 h of co‐culture, TCMK‐1 cells were cultured in MEM containing 100 μg/mL COM for an additional 24 h. The cells were then thoroughly washed with PBS three times to remove unbound COM crystals. Finally, images were captured using a bright field inverted microscope, and the volume of adherent crystals in at least 10 randomized high‐power fields per well was counted.

### 2.6 Elisa measurement

Supernatants from Mφ‐Vec and Mφ‐IL10 macrophages were collected for IL‐10 level determination, while supernatants from mouse kidney homogenates were utilized to assess the levels of IL‐10, IL‐1*β*, IL‐6, and TNF‐*α* using enzyme‐linked immunosorbent assay (ELISA) kits (Invitrogen, USA). All ELISA procedures were conducted in strict accordance with the manufacturer's instructions. The experiments were performed in triplicate, and the results were expressed as the mean values.

### 2.7 Quantitative PCR analysis

Total RNA from cells and tissues was extracted utilizing the FastPure Cell/Tissue Total RNA Isolation Kit (RC101, Vazyme, China) following the manufacturer's instructions, and RNA concentrations were quantified using a Thermo NanoDrop 2000 spectrophotometer. Total RNA (500 ng) was reverse‐transcribed into cDNA following the protocols provided with the HiScript III RT SuperMix (RC323, Vazyme, China). Quantitative real‐time PCR (qPCR) was conducted on an ABI 7500 Real‐Time PCR System (Applied Biosystems) with SYBR qPCR Master Mix (Q712, Vazyme, China). Gene transcript levels were calculated using the 2^−ΔΔ*CT*^ method, with 18S rRNA serving as the endogenous reference control. Primer used as following: Il‐10, Forward (F): 5′‐GCTCTTACTGACTGGCATGAG‐3′, Reverse (R): 5′‐CGCAGCTCTAGGAGCATGTG‐3′; Ym‐1, F: 5′‐CAGGTCTGGCAATTCTTCTGAA‐3′, R: 5′‐GTCTTGCTCATGTGTGTAAGTGA‐3′; Arg‐1, F: 5′‐CTCCAAGCCAAAGTCCTTAGAG‐3′, R: 5′‐AGGAGCTGTCATTAGGGACATC‐3′; Il‐6, F: 5′‐TAGTCCTTCCTACCCCAATTTCC‐3′, R: 5′‐TTGGTCCTTAGCCACTCCTTC‐3′; IL‐1*β*, F: 5′‐ATCCAGCTTCAAATCTCGC‐3′, R: 5′‐ATCTCGGAGCCTGTAGTGC‐3′; Tnf‐*α*, F: 5′‐CCCTCACACTCAGATCATCTTCT‐3′, R: 5′‐GCTACGACGTGGGCTACAG‐3′; Ccl2, F: 5′‐TTAAAAACCTGGATCGGAACCAA‐3′, R: 5′‐GCATTAGCTTCAGATTTACGGGT‐3′; 18S rRNA, F: 5′‐TGTGCCGCTAGAGGTGAAATT‐3′, R: 5′‐TGGCAAATGCTTTCGCTTT‐3′.

### 2.8 PAS staining, HE staining and polarized light optical microscopy

Periodic acid schiff (PAS) staining was performed according to the manufacturer's instructions (Solarbio). Hematoxylin–eosin (HE) staining was performed on kidney sections according to standard procedures. Then, the sections were photographed using the TissueFAXS system and AX10 polarized light optical microscope (Zeiss, Germany).

### 2.9 Immunostaining

Kidney samples embedded in paraffin were sectioned into 5‐μm slices, followed by deparaffinization and dehydration. For immunohistochemical staining, the sections were incubated with primary antibodies targeting OPN (1:200, 22952‐1‐AP, Proteintech), IL‐10 (1:200, 60269‐1‐Ig, Proteintech), and IL‐1*β* (1:100, 12242, Cell signal technology). The sections were subsequently counterstained with hematoxylin. Immunofluorescence staining was performed using an anti‐F4/80 antibody (1:200, ab‐6640, Abcam), and IL‐10 (1:200, 60269‐1‐Ig, Proteintech) was used. Nuclei were visualized by staining with Antifade Mounting Medium containing DAPI (P0131, Beyotime, China). All slides were captured using panoramic imaging. Scoring and counting were conducted on 10 randomly selected high‐power fields (HPF, ×200).

### 2.10 Histopathological observation and kidney injury scores

Two blinded pathologists evaluated the severity of renal injury based on specific histological criteria: tubular dilation, presence of urinary casts, brush border loss, and inflammatory cell infiltration. For this assessment, each renal tubule within the 10 randomly selected HPF was assigned one point for each observed feature. The cumulative score for each mouse was then determined. The inflammatory area was defined as the region infiltrated by inflammatory cells and was characterized by the percentage of HPF that it occupies. Concentrations of blood urea nitrogen (BUN) and serum creatinine were measured using urea or creatinine assay kit, respectively (Changchun Huili Bioengineering Institute, China).

### 2.11 Flow cytometry

Renal cells were isolated using a 40‐μm cell strainer (Solarbio, China). Following washing with PBS, the cells were stained with flow cytometry antibodies to detect CD45 (103247, BioLegend), CD86 (105029, BioLegend), CD206 (141717, BioLegend), and F4/80 (123149, BioLegend), in accordance with the manufacturer's instructions. Data acquisition was performed using a FACS Aria II flow cytometer (BD Biosciences), and analysis of the results was conducted using FlowJo software (Version X).

### 2.12 Calcein‐Propidium iodide staining

Calcein‐PI staining was performed using a calcein/PI Cell assay kit (Beyotime, China). Briefly, after treating TCMK‐1 cells with COM as described, the medium was removed, and then an appropriate amount of calcein/PI working solution was added and incubated at 37°C for 30 min in the dark. Images were captured using a fluorescence microscope (Zeiss, Germany).

### 2.13 Detection of ROS

Intracellular ROS were detected using dihydroethidium (DHE; Beyotime, China) fluorescent probes according to the manufacturer's instructions. Briefly, TCMK‐1 cells were seeded in a 24‐well plate and treated with COM as described, then incubated with 10 μM DHE at 37°C for 30 min. Fluorescence was then measured on a Clariostar microplate reader (BMG Labtech, Offenburg, Germany) at 488 nm excitation and 525 nm emission.

### 2.14 LDH release assay

Lactate dehydrogenase (LDH) release was detected using an LDH assay kit (Beyotime, China) according to the manufacturer's instructions. Briefly, TCMK‐1 cells were seeded in a 24‐well plate and treated with COM as described. The supernatant was then collected as a control. After sufficient washing with PBS, the cells were treated with 1.5% Triton X‐100. The cells and supernatant were incubated with coenzyme I and 2, 4‐dinitrophenylhydrazine for 30 min at 37°C. Absorbance was then measured in a Clariostar microplate reader at 490 nm.

### 2.15 Statistical analysis

All analyses were performed using GraphPad (GraphPad Prism 9.0, San Diego, CA, US). Data were presented as the mean ± standard error of the mean (S.E.M.). Statistical comparisons between two groups were conducted using the unpaired Student's *t*‐test. For comparisons involving multiple groups, analysis of variance was employed, followed by Tukey's post‐hoc test. The results were considered significant at * *p* < 0.05, ** *p* < 0.01, ***p < 0.001, and ****p < 0.0001.

## 3 RESULTS

### 3.1 Construction of engineered macrophage secreting IL‐10

**FIGURE 1:**
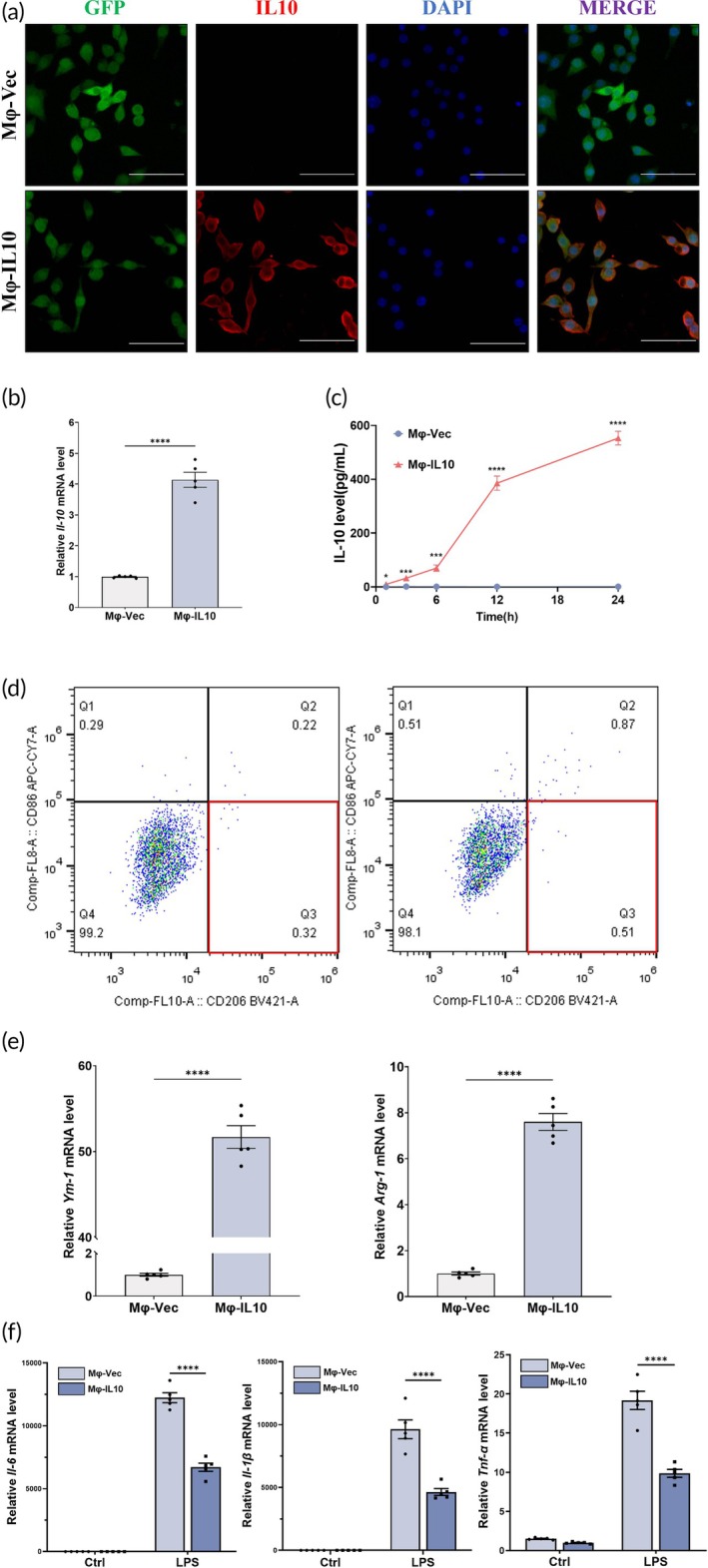
Establishment and characterization of engineered macrophage secreting IL‐10(Mφ‐IL10). (a) Representative images of the engineered macrophage stained with IL‐10. Bar = 50 μm. (b) qPCR analysis of IL‐10 mRNA in engineered macrophage. (c) IL‐10 concentration of engineered macrophage medium. Data are expressed as mean ± SD; statistical significance was determined by two‐way ANOVA with the Tukey multiple comparison test. *p < 0.05, **p < 0.01, ****p < 0.0001. (d) Flow cytometry analysis of engineered macrophage(CD86, M1 cell surface marker, and CD206, M2 cell surface marker). (e) qPCR analysis of Ym‐1 and Arg‐1 mRNA in engineered macrophage. (f) qPCR analysis of IL‐6, IL‐1*β*, and TNF‐*α* mRNA in engineered macrophage treated with/without LPS.

Considering the anti‐inflammatory effects and macrophage‐polarizing properties of IL‐10, we generated RAW264.7 macrophages that constitutively secrete IL‐10 using lentiviral vectors (referred to as Mφ‐IL10) (Figure 1a–d). IL‐10 expression was verified through immunofluorescence (Figure 1a), quantitative real‐time PCR (qRT‐PCR) (Figure 1b), and ELISA (Figure 1c). Flow cytometry was utilized to assess the polarization status of Mφ‐vec and Mφ‐IL10 (Figure 1d). In comparison to Mφ‐vec, Mφ‐IL10 exhibited significantly higher levels of IL‐10 (Figure 1a), the expression of IL‐10 mRNA (Figure 1b) and the concentration of IL‐10 in the supernatant of Mφ‐IL10 was higher (552.3 pg/mL vs. 1 pg/mL) (Figure 1c). The potential auto‐polarization effect of Mφ‐IL10 was also taken into account. Flow cytometry results showed that the percentage of M2 phenotype (CD206^+^CD86^−^) was not significantly elevated in the Mφ‐IL10 group (Figure 1d). These findings confirm the successful generation of IL‐10‐secreting macrophages and demonstrate that the expressed IL‐10 does not self‐direct polarization.

### 3.2 Mφ‐IL10 induces M2 polarization and suppresses inflammatory cytokines in vitro

Subsequently, we evaluated the M2‐polarizing influence of Mφ‐IL10 in vitro. The murine monocyte/macrophage cell line RAW264.7 was co‐cultured with engineered macrophages using transwell systems. Notably, the mRNA levels of Ym‐1 and Arg‐1 in RAW264.7 cells co‐cultured with Mφ‐IL10 were significantly elevated compared to those co‐cultured with Mφ‐vec, validating the M2‐polarizing capacity of Mφ‐IL10 (Figure 1e). Furthermore, we assessed the anti‐inflammatory effects of Mφ‐IL10. Compared to RAW264.7 cells co‐cultured with Mφ‐vec, the expression levels of IL‐6, TNF‐*α*, and IL‐1*β* were markedly reduced in those co‐cultured with Mφ‐IL10 (Figure 1f). Collectively, these findings indicate that IL‐10 expression in Mφ‐IL10 can facilitate the polarization of M0 macrophages toward the M2 phenotype and suppress the expression of inflammatory cytokines.

### 3.3 Mφ‐IL10 transplantation mitigates nephrocalcinosis‐induced weight loss and renal dysfunction

**FIGURE 2:**
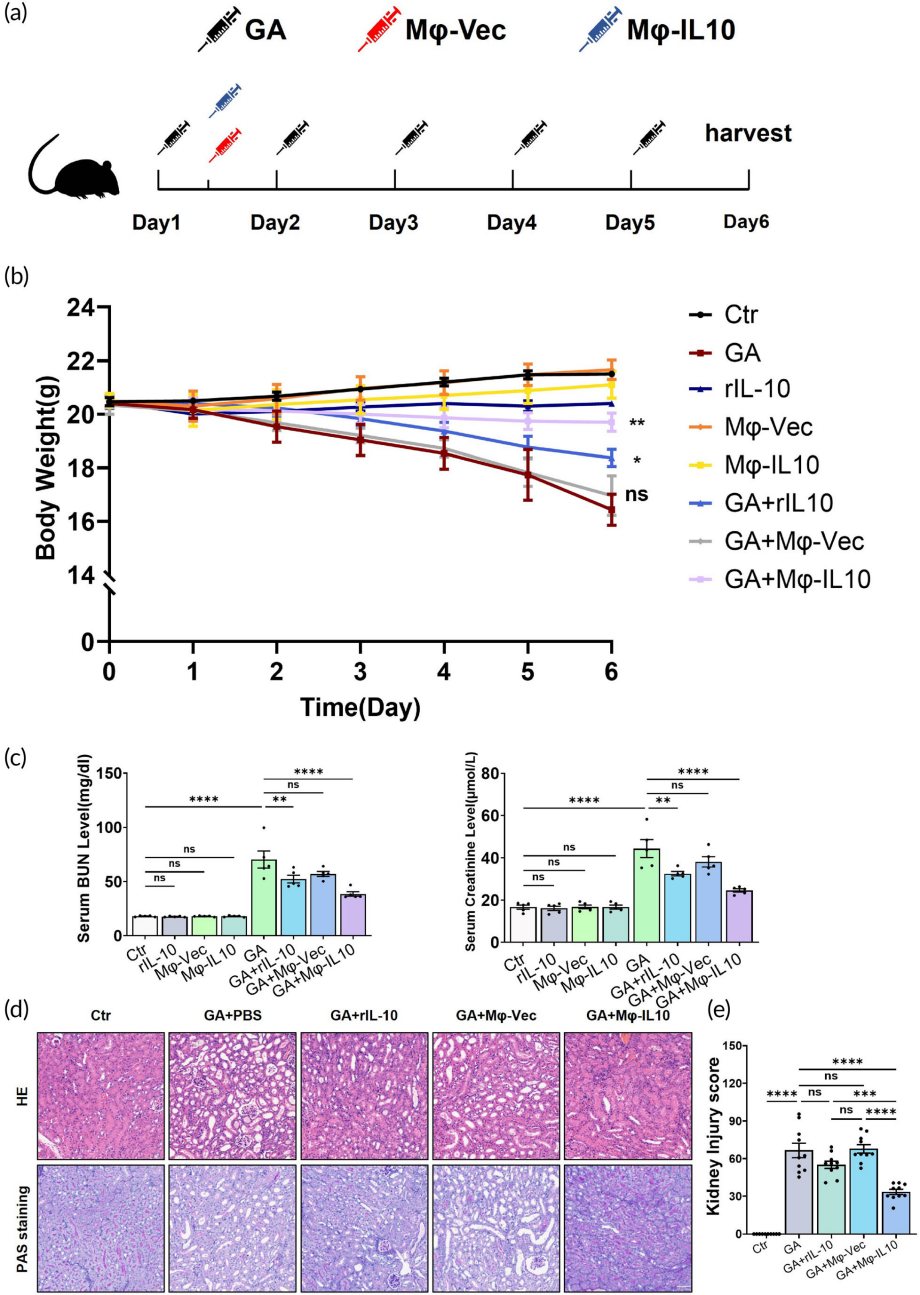
Mφ‐IL10 transplantation attenuates renal tubular injury in vivo. (a) Diagram of the animal experimental design. 18–20 g male C57BL/6 mice were randomly divided into 8 groups (*n* = 5). (b) Mouse body weight of each group during the experiment process (*n* = 5). (c) Level of blood urea nitrogen and concentrations of serum creatinine when sacrificed. (d) Representative images of HE and PAS staining of kidney tissue. Bar = 50 μm. (e) Score of tubular injury evaluated via tubular dilation, disappearance of brush border, and enlargement of renal interstitial matrix. Data are presented as mean ± SEM, *p < 0.05, **p < 0.01, ***p < 0.001, ****p < 0.001.

Relying on our previous experience with animal models of CaOx formation, we implemented a stable and reliable method for inducing CaOx formation in an animal model. This was achieved through the intraperitoneal injection of 75 mg/kg body weight of glyoxylic acid (GA) administered over six consecutive days (Figure 2a). Subsequently, PBS, engineered macrophages, or recombinant mouse IL‐10 were injected into the mice via the tail vein 10 h post‐GA administration. To assess the safety and efficacy of Mφ‐IL10 transplantation, we monitored the body weight of each mouse daily, commencing prior to the initiation of GA injections (Figure 2b). The weight records revealed that Mφ‐IL10 transplantation significantly mitigated weight loss associated with GA injections compared to treatments with rIL‐10 and Mφ‐vec transplantation. Additionally, neither rIL‐10 injections nor macrophage transplantation alone resulted in mortality or weight loss in the mice. Serum levels of BUN and creatinine were indicative of Mφ‐IL10's ability to ameliorate the decline in renal function induced by nephrocalcinosis (Figure 2c). Similarly, neither rIL‐10 injections nor macrophage transplantation alone led to an increase in BUN and creatinine levels. We also evaluated the distribution and viability of Mφ‐IL10 in the liver, lungs, and kidneys at day 7 post‐injection (Supplementary Figure 1). Findings indicate that Mφ‐IL10 can survive long‐term in multiple organs without causing embolism. These results confirm the safety and efficacy of macrophage transplantation therapy.

### 3.4 Mφ‐IL10 transplantation ameliorates renal tubular injury induced by nephrocalcinosis

Kidney tubular cells are pivotal in the pathogenesis of nephrocalcinosis; they are not only primarily affected by crystal exposure but also contribute to the exacerbation of crystal‐induced injury, creating a vicious cycle. We assessed renal tubular injury using histological indices, which included the loss of tubular cells, the absence of brush border, and the dilation of tubular lumen as observed in H&E and PAS staining sections (Figure 2d). Transplantation with Mφ‐IL10 mitigated the elevated tubular injury scores induced by crystal formation.

### 3.5 Mφ‐IL10 transplantation suppresses the deposition and adhesion of crystal in the mouse model of nephrocalcinosis

**FIGURE 3:**
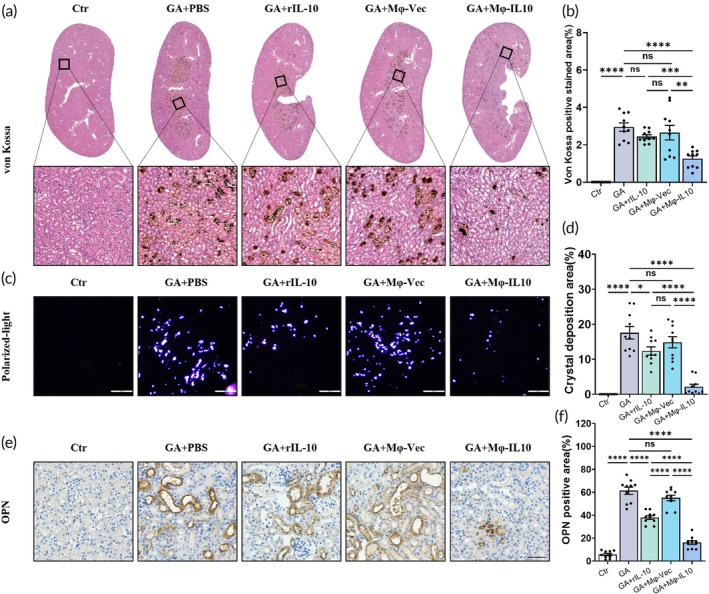
Mφ‐IL10 transplantation suppresses the deposition and adhesion of stone in the mouse model of nephrolithiasis. (a) Representative images of Von Kossa staining in the kidney tissue. Scale bar = 50 μm. (b) The statistical analyses of Von Kossa stain positive area. (c) Representative images of renal CaOx crystal deposition via polarized light microscopy (scale bar = 200 μm). (d) The statistical analyses of CaOx crystal deposition. (e) Representative images of the immunohistological staining of OPN (scale bar = 50 μm). (f) The statistical analyses of OPN stain positive area. Data are presented as mean ± SEM, *p < 0.05, **p < 0.01, ****p < 0.001.

Crystal deposition and adhesion are hallmark features of nephrocalcinosis. We assessed crystal deposition in mouse models of GA‐induced nephrocalcinosis. Von Kossa staining revealed reduced renal calcium salt deposition in the Mφ‐IL10 transplantation group relative to the groups treated with GA, rIL‐10, and Mφ‐vec transplantation (Figure 3a, b). Correspondingly, polarized light microscopy results indicated that Mφ‐IL10 transplantation significantly reduced crystal deposition in kidney tissues (Figure 3c, d). Osteopontin (OPN), a secreted glycoprotein that is upregulated in response to renal damage, particularly in the context of crystal‐induced injury, was evaluated for its expression in our nephrocalcinosis mouse model. Immunohistochemical staining showed that the GA‐induced upregulation of OPN was mitigated by Mφ‐IL10 transplantation (Figure 3e, f).

### 3.6 Mφ‐IL10 transplantation attenuates crystal‐induced inflammation in the kidney

**FIGURE 4:**
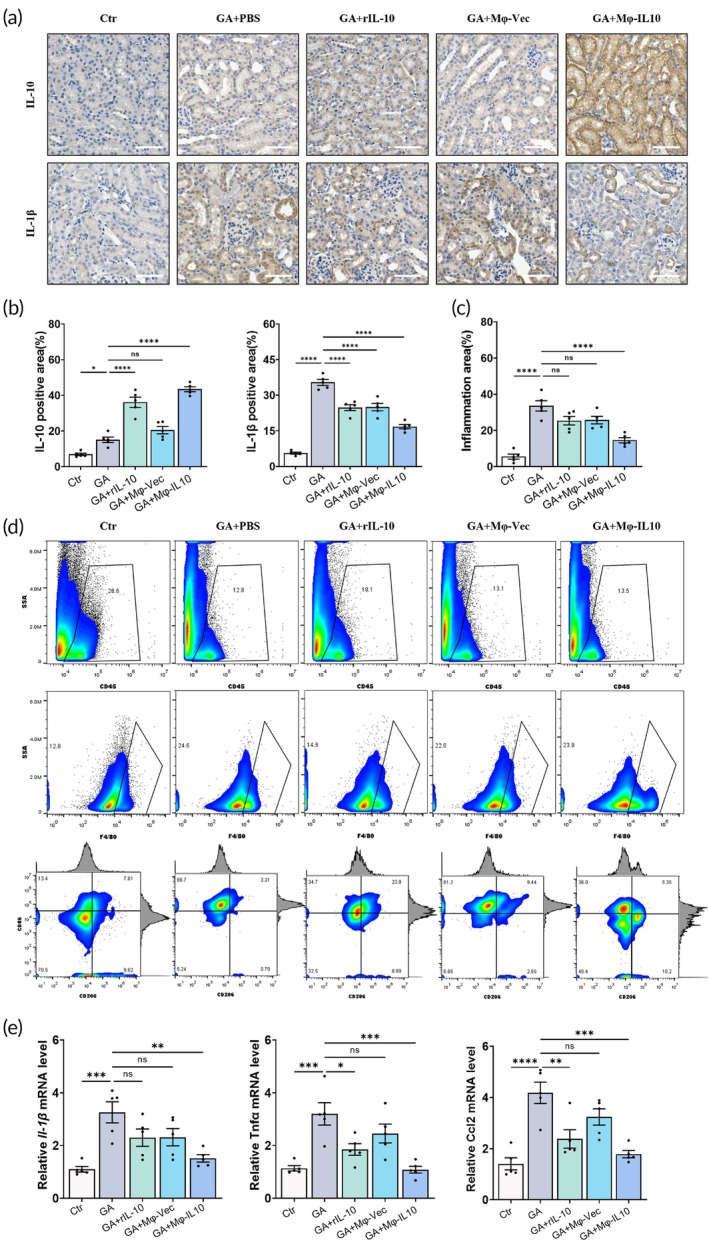
Mφ‐IL10 transplantation attenuates crystal‐induced inflammation in kidney. (a) Representative immunohistochemical staining of IL‐10 and IL‐1*β* in the mice kidney tissue. Scale bar = 50 μm. (b) The statistical analyses of IL‐10 and IL‐1*β* stain positive area. (c) The statistical analyses of inflammatory cell infiltration in the PAS staining section. (d) Flow cytometry analysis of immunocyte in the mice kidney tissue. (e) Results of qPCR assay of IL‐1*β*, Tnf‐*α*, and Ccl2 mRNA expression in the kidney tissues. Data are presented as mean ± SEM, *p < 0.05, **p < 0.01, ****p < 0.001.

Prior research has established that macrophages are crucial in the formation and associated injury of nephrocalcinosis. Based on the in vitro polarizing effects of Mφ‐IL10 on macrophages, we hypothesize that Mφ‐IL10's protective role may be mediated by reducing inflammatory responses through the polarization of macrophages in vivo. To test this hypothesis, we evaluated inflammatory cell infiltration in renal tissues. Quantitative analysis of PAS staining revealed that the Mφ‐IL10 transplantation group exhibited reduced inflammatory cell infiltration compared to the GA treatment control group, the rIL‐10 treatment group, and the Mφ‐vec transplantation group (Figures 2d and 4b). Furthermore, flow cytometry confirmed the resolution of inflammatory cell infiltration in the Mφ‐IL10 transplantation group. Analysis of macrophage subsets indicated a lower M1/M2 ratio in the Mφ‐IL10 group, thereby confirming the in vivo M2 polarizing capability of Mφ‐IL10 (Figure 4d). Additionally, immunohistochemical staining assessed the levels of inflammatory factors in the kidney, demonstrating that the pro‐inflammatory cytokine IL‐1*β* was reduced in the Mφ‐IL10 transplantation group relative to other groups (Figure 4a, c), while the anti‐inflammatory cytokine IL‐10 was most abundant in this group due to Mφ‐IL10 transplantation (Figure 4a, c). We also quantified mRNA expression levels of inflammatory cytokines TNF‐*α*, IL1‐*β*, and CCL‐2, and our data indicate that Mφ‐IL10 transplantation suppresses renal inflammatory infiltration in vivo.

### 3.7 Mφ‐IL10‐mediated macrophage M2 polarization promotes COM crystal clearance in vitro

**FIGURE 5:**
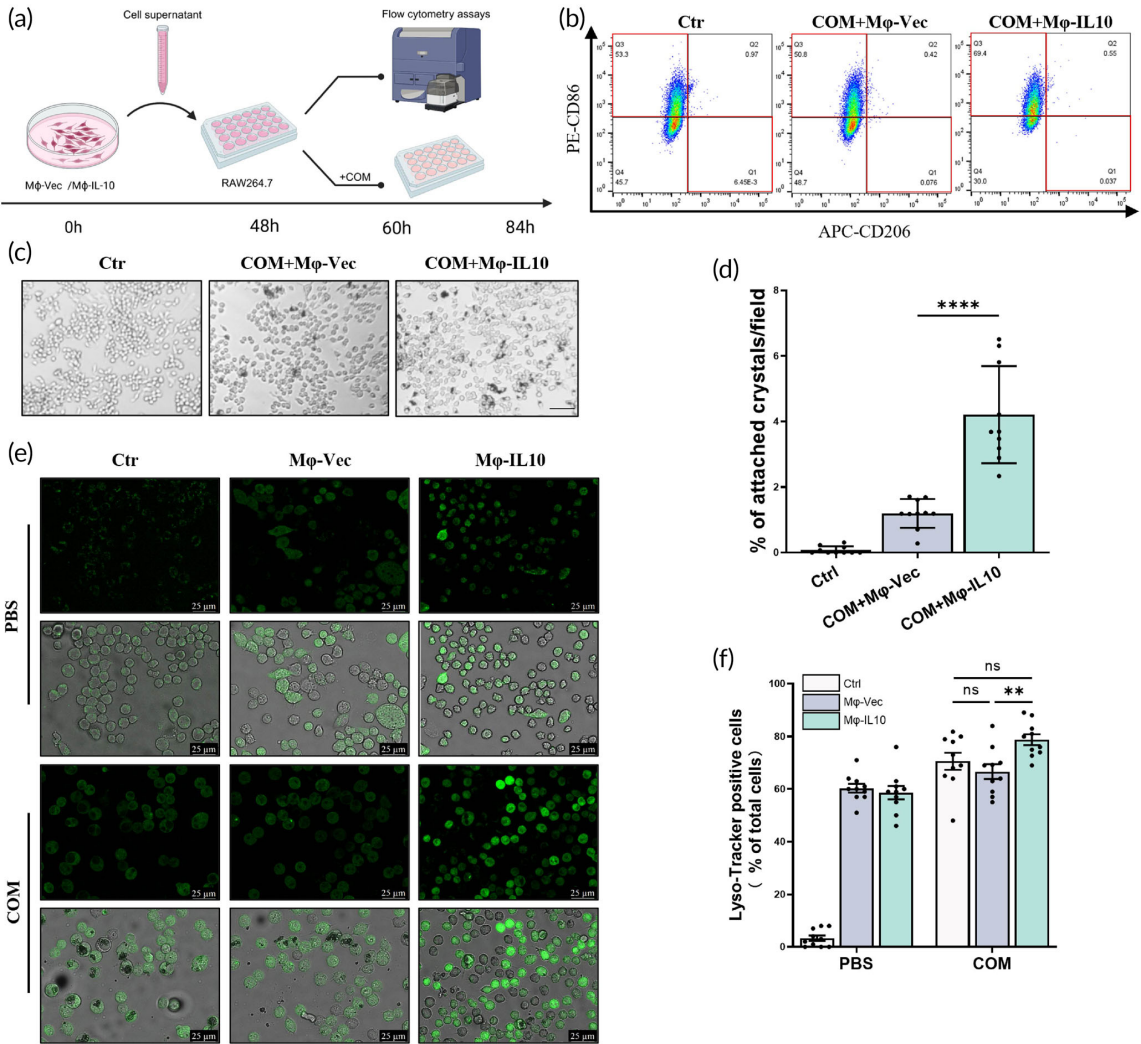
Mφ‐IL10‐mediated macrophage M2 polarization promotes COM crystal clearance in vitro. After 48 h of incubation, supernatants of engineered macrophages were collected and used to induce polarization of RAW264.7 cells. (a) Diagram of the cell experimental design. (b) Flow cytometry results of engineered macrophages on polarization of RAW264.7 cells. (c) and (d) Representative images of the fields under the microscope and the results of statistical analyses. Scale bar = 50 μm. (e) and (f) Representative images and statistical analyses of Lyso‐Tracker Green staining. Data are present as mean ± SEM, *p < 0.05, **p < 0.01, ****p < 0.001.

To investigate the protective mechanisms of Mφ‐IL10 transplantation in renal injury induced by CaOx, we utilized the RAW264.7 cell line as an in vitro model. After a 48‐h culture period with Mφ‐vec or Mφ‐IL10, we collected the supernatant and applied it to RAW264.7 cells to induce differentiation for 12 h (Figure 5a). Subsequent flow cytometry analysis indicated that RAW264.7 cells treated with the Mφ‐IL10 supernatant had a higher percentage of CD86^+^ cells compared to those treated with the Mφ‐vec supernatant (Figure 5b). Moreover, these Mφ‐IL10‐treated RAW264.7 cells demonstrated an increased adherence to COM crystals (Figure 5c, d). Lyso‐Tracker positivity in the cells was regarded as indicative of acidic lysosome activation. COM crystal stimulation increased the proportion of Lyso‐Tracker‐positive cells, and the induction with Mφ‐IL10 supernatant further amplified this effect, both in terms of proportion and fluorescence intensity (Figure 5e, f).

### 3.8 Mφ‐IL10 transplantation protects renal tubular cells from COM toxicity and adhesion

**FIGURE 6:**
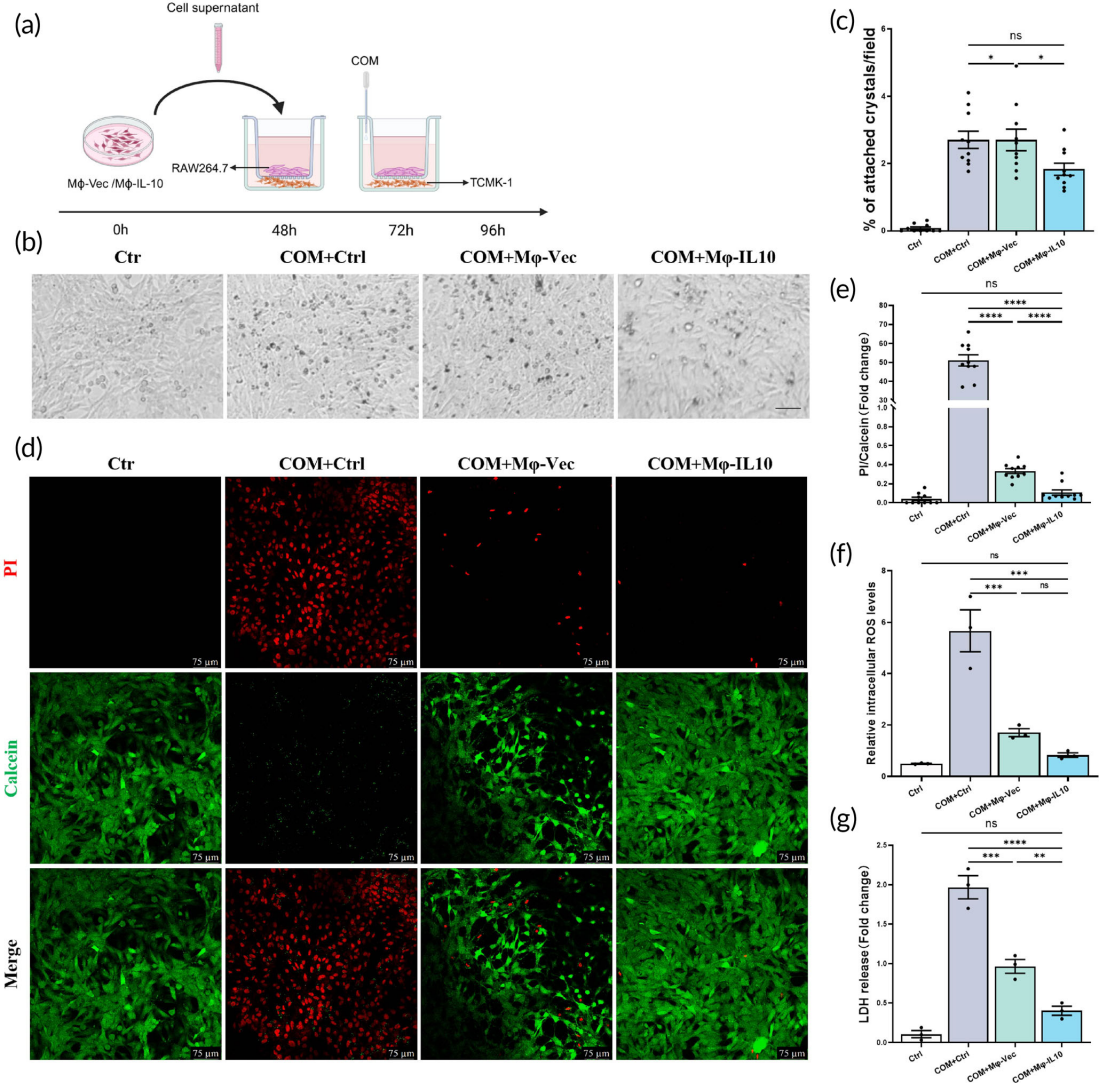
Mφ‐IL10 attenuates crystal‐induced oxidative damage via modulating macrophage M2 polarization in vitro. (a) Diagram of the co‐culture experimental design. (b) and (c) Representative images of the fields under the microscope and the results of statistical analyses. Scale bar = 50 μm. (d) Representative images and statistical analyses of calcein‐propidium iodide (PI) staining. Scale bar = 75 μm. (e) Statistical analyses on the ratio of PI/calcein positive cells. (f) DHE assay for intracellular ROS. (g) The statistical analyses of IL‐10 stain positive area. Data are presented as mean ± SEM, *p < 0.05, **p < 0.01, ***p < 0.001, ****p < 0.001.

To further elucidate the protective mechanism of Mφ‐IL10‐mediated macrophage M2 polarization in COM‐induced renal injury, we co‐cultured immortalized mouse renal tubular epithelial cells TCMK‐1 and RAW264.7 cells as an in vitro model of COM‐induced renal injury (Figure 6a). After treating RAW264.7 cells with supernatant as described previously, they were placed in the upper chamber and co‐cultured with TCMK‐1 in the lower chamber for 24 h. Subsequently, COM solution was added to the lower chamber to continue incubation for an additional 24 h. We also performed an in vitro adhesion assay and found that transplantation of Mφ‐IL10 reduced the volume of adherent crystals on TCMK‐1 cells (Figure 6b, c). A Calcein‐PI staining assay showed that COM induced an increase in TCMK‐1 cell death, and Mφ‐IL10 transplantation treatment blocked this effect (Figure 6d, e).

### 3.9 Mφ‐IL10 transplantation decreased the reactive oxygen species (ROS) induced by COM crystals in vitro

It is well known that oxidative damage is largely involved in the pathogenesis of nephritis. Therefore, we evaluated the effect of Mφ‐IL10 transplantation on ROS levels in renal epithelial cell injury induced by calcium oxalate. The DHE assay showed an increase in intracellular ROS in the COM group compared with the control group (Figure 6f). Mφ‐IL10 significantly attenuated the COM‐induced ROS upregulation. LDH release assay showed similar results (Figure 6g).

## 4 DISCUSSION

Macrophages, integral to both calcium oxalate (CaOx) crystal formation and dissolution, represent a promising therapeutic target for nephrocalcinosis. In this study, we generated engineered macrophages capable of expressing and secreting the anti‐inflammatory cytokine IL‐10 and observed that a single transplantation of these macrophages exerted significant therapeutic effects in a murine model of CaOx. To our knowledge, this is the inaugural report on the application of macrophage transplantation in the treatment of nephrocalcinosis.

IL‐10, an anti‐inflammatory cytokine produced by activated immune cells,^20^ primarily targets macrophages.^21^ The functionality of macrophages is contingent upon metabolic processes and ROS signaling.^22^ However, excessive ROS production can induce oxidative stress, leading to cellular dysfunction.^23^ IL‐10 is crucial for curbing excessive ROS production in macrophages,^24^ and it also promotes M2 macrophage polarization, which can engulf crystals and thus prevent crystals formation.^25^ Based on these findings, IL‐10 theoretically holds therapeutic potential for inflammatory diseases. However, the application of IL‐10 in various diseases has yielded mixed results, with similar achievements and challenges.^26^, ^27^, ^28^ Inflammatory bowel disease (IBD) stands out as the most extensively studied condition in terms of IL‐10 therapy.^29^, ^30^, ^31^, ^32^, ^33^ While recombinant IL‐10 or IL‐10 overexpression has shown benefits in animal models and some clinical trials,^34^, ^35^, ^36^ it has not universally improved patient outcomes.^35^, ^37^ The low local enrichment of IL‐10 and its systemic effects' duality may underpin these barriers. Several delivery vectors, including IL‐10‐expressing Lactococcus or Bifidobacterium, have demonstrated efficacy in animal models.^34^ Subsequent improvements have also enhanced clinical scores in Phase I trials for patients with Crohn's disease.^38^, ^39^

Similarly, IL‐10 has been implicated in a myriad of renal diseases through its activation of anti‐inflammatory responses, immune regulation, and mitigation of renal tissue fibrosis.^26^ However, the use of IL‐10 in human therapy requires further investigation.^40^, ^41^, ^42^ Several novel drug delivery systems containing IL‐10 have shown promise in improving clinical outcomes in renal diseases. Tang et al. prepared IL‐10‐laden extracellular vesicles (EVs) using RAW264.7 cells and found that these significantly ameliorated renal tubular injury and inflammation induced by ischemia/reperfusion injury.^43^ They discovered that IL‐10‐laden EVs target tubular epithelial cells and effectively drive macrophage M2 polarization by engaging with macrophages in the tubulointerstitium. Similarly, Soranno et al. improved renal outcomes after ischemic acute kidney injury (AKI) by delivering IL‐10 via hydrogel injection.^44^

Macrophage‐based therapy offers distinct advantages. First, macrophages can survive and function in a compatible tissue microenvironment with a single injection required. Engineered macrophages, being regulated by the immune system, can avoid the risk of overdose associated with direct IL‐10 cytokine therapy.^45^ Moreover, macrophages accumulate at crystal deposition sites and secrete inflammatory and chemokines in response to CaOx, recruiting additional macrophages.^15^, ^46^ Second, engineered macrophages interact with the local microenvironment, modulating their biological behavior for enhanced tissue compatibility. In our study, we observed higher concentrations of engineered macrophages in the kidneys of the stone group, particularly around CaOx crystals. These characteristics, combined with precise secretory capabilities, render macrophages effective cytokine delivery vectors.

In this study, we demonstrated that transplantation of engineered macrophages overexpressing IL‐10 effectively cleared CaOx crystals and mitigated CaOx‐induced renal tubular injury in mice. We measured IL‐10 concentrations in the kidneys within 72 h post‐injection and plotted the change curve. Compared to the rapid increase and decline observed in the rIL‐10 group, IL‐10 levels in the Mφ‐IL10 group increased smoothly and persisted over time. Given that subsequent experiments revealed Mφ‐IL10 cells were still viable in kidney tissue on Day 6, this indirectly confirms that IL‐10 secretion by Mφ‐IL10 is modulated by the immune environment. Although no significant difference in macrophage numbers was observed between the rIL‐10 and Mφ‐IL10 groups, the M1/M2 ratio varied significantly, which may explain how IL‐10 can simultaneously enhance crystal clearance and inhibit inflammation‐related injury.

Our study has limitations, including a constrained observation period, an absence of comparisons between various injection regimens, and long‐term safety assessments of the treatment. Additionally, this study did not address the distribution, migration, and life cycle of transplanted macrophages in vivo. Moreover, CaOx crystal formation under experimental conditions has a defined starting point, which is not feasible in clinical settings. Therefore, further experiments are warranted to determine the duration of engineered macrophages and their therapeutic effects at different stages of crystal formation. Lastly, considering the potential side effects of long‐term IL‐10 exposure and systemic effects, the precision of targeting and switching of engineered macrophages should be further refined.

## AUTHOR CONTRIBUTIONS

Haixing Mai, Xu Zhang, and Luo Zhang supervised the research, contributed to the overall concept and design of the study, and provided critical feedback instrumental in shaping the research, analysis, and manuscript preparation. Wenlei Zhao, Kailong Wang, and Junnan Xu conducted the studies, performed the experiments, analyzed the data, and drafted the manuscript. Lequan Wen, Yan Wu, and Hairui Chen carried out specific experiments and contributed to the writing of the manuscript. Weihao Chen, Haitao Liu, and Yanzhong Liu offered guidance on experimental design, provided analytical tools, and reviewed and edited the manuscript. All authors reviewed and approved the final manuscript.

## FUNDING INFORMATION

This work was supported by the Natural Science Foundation of China (No. 82173259), the Natural Science Foundation of Beijing (No. 7222232), Science and Technology Program of Beijing (Z221100007422123), Chinese PLA General Hospital Youth Independent Innovation Science Fund Support Project (No. 22QNFC035, No. 22QNFC045).

## CONFLICT OF INTEREST STATEMENT

All authors declare no conflicts of interest.

## Supporting information


**Supplementary Figure 1.** The distribution and survival of Mφ‐IL10 in the liver, lung, and kidney at day 7.

## Data Availability

All data generated or analysed during this study are included in this published article.
