# Supplementary figures and images for: Revolutionizing nephrocalcinosis treatment: IL‐10 engineered macrophages as a novel therapeutic approach
Source: Bioeng Transl Med. 2025 Jul 10;11(1):e70047. doi: 10.1002/btm2.70047 (PMC12821214; doi:10.1002/btm2.70047)

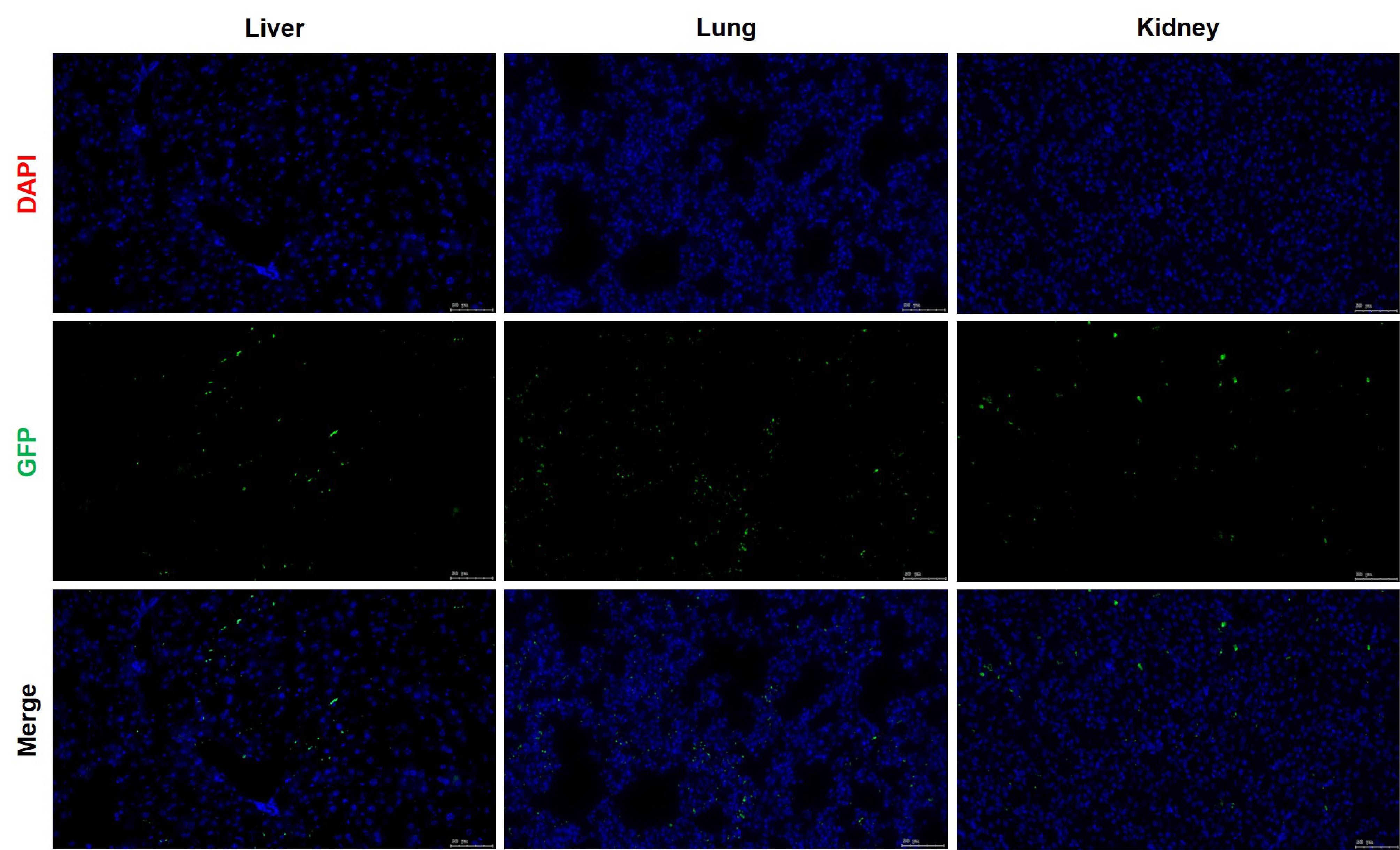

Supplement: Supplementary file 1 — Supplementary Figure 1. The distribution and survival of Mφ‐IL10 in the liver, lung, and kidney at day 7. [file BTM2-11-e70047-s001.jpg]
